# Global identification and analysis of long non-coding RNAs in diploid strawberry *Fragaria vesca* during flower and fruit development

**DOI:** 10.1186/s12864-015-2014-2

**Published:** 2015-10-19

**Authors:** Chunying Kang, Zhongchi Liu

**Affiliations:** Department of Cell Biology and Molecular Genetics, University of Maryland, College Park, MD 20742 USA; College of Horticulture and Forestry Sciences, Huazhong Agricultural University, Wuhan, 430070 China

**Keywords:** LncRNA, Strawberry, Flower, Fruit, RNA-seq, *Fragaria vesca*, *Rosaceae*

## Abstract

**Background:**

Long non-coding RNAs (lncRNAs) are a new class of regulatory molecules with roles in diverse biological processes. While much effort has been invested in the analysis of lncRNAs from established plant models *Arabidopsis*, maize, and rice, almost nothing is known about lncRNAs from fruit crops, including those in the *Rosaceae* family.

**Results:**

Here, we present a genome-scale identification and characterization of lncRNAs from a diploid strawberry*, Fragaria vesca*, based on rich RNA-seq datasets from 35 different flower and fruit tissues. 5,884 Fve-lncRNAs derived from 3,862 loci were identified. These lncRNAs were carefully cataloged based on expression level and whether or not they contain repetitive sequences or generate small RNAs. About one fourth of them are termed high-confidence lncRNAs (hc-lncRNAs) because they are expressed at a level of FPKM higher than 2 and produce neither small RNAs nor contain repetitive sequence. To identify regulatory interactions between lncRNAs and their potential protein-coding (PC) gene targets, pairs of lncRNAs and *PC* genes with positively or negatively correlated expression trends were identified based on their expression; these pairs may be candidates of *cis-* or *trans*-acting lncRNAs and their targets. Finally, blast searches within plant species indicate that lncRNAs are not well conserved.

**Conclusions:**

Our study identifies a large number of tissue-specifically expressed lncRNAs in *F. vesca*, thereby highlighting their potential contributions to strawberry flower and fruit development and paving the way for future functional studies.

**Electronic supplementary material:**

The online version of this article (doi:10.1186/s12864-015-2014-2) contains supplementary material, which is available to authorized users.

## Background

With the advent of new genomic techniques such as tiling arrays and next generation sequencing [1, 2], non-coding RNAs are increasingly identified and recognized as an integral and functional component of the genome. While some non-coding RNAs perform housekeeping functions, such as tRNAs, rRNAs and small nuclear RNAs, others such as microRNAs and small interfering RNAs (siRNA) play critical regulatory roles during development or stress responses [3, 4]. Non-coding RNAs with a length greater than 200bp are defined as long non-coding RNAs (lncRNAs). The lncRNAs can be grouped into three subclasses: 1) long non-coding natural antisense transcripts (lnc-NATs), 2) intronic lncRNAs, and 3) intergenic lncRNAs. Like protein-coding (PC) genes, a majority of lncRNAs are transcribed by RNA polymerase II with a 5′ cap and a 3′ poly-A tail in animals [5]. In plants, however, lncRNAs can be transcribed by PolII, IV, and V, therefore some may lack poly-A tails [6–9].

A growing number of reports revealed lncRNAs from animals, especially human; they are involved in diverse biological processes, such as development, cellular differentiation, and diseases including cancers. LncRNAs may serve as diagnostic markers or even therapeutic targets [10–15]. Studies of animal lncRNAs showed that lncRNAs function through a number of mechanisms. First, lncRNAs act in epigenetic regulation of gene expression. For instance, the mammalian *XIST RNA* initiates X chromosome inactivation *in cis* to equalize gene expression between males and females [16, 17]. HOTAIR (HOX antisense intergenic RNA) is able to mediate transcriptional repression of *HOX* loci *in trans* by modulating histone methylation [18]. Second, lncRNAs may directly interact with proteins to titrate their functions and are thus called ‘decoys’. A well-known example is the lncRNA named TERRA, which was demonstrated to be the natural ligand and inhibitor of telomerase [19]. Third, lncRNAs may act as a scaffold to form a complex with other proteins. Together, lncRNAs were demonstrated to play bona fide and essential roles in animals.

In contrast, there are only a handful of reports on the functions of lncRNAs in plants. FLOWERING LOCUS C (FLC) is a key flowering repressor in the vernalization pathway. To ensure epigenetic silencing of *FLC*, a lnc-*NAT* named *COOLAIR* (*COLD INDUCED LONG ANTISENSE INTRAGENIC RNA*) and an intronic lncRNA called *COLDAIR* (*COLD ASSISTED INTRONIC NONCODING RNA*) could be induced after vernalization treatment to gradually repress the expression of *FLC* by promoting methylation [7, 20]. Another intergenic lncRNA induced by phosphate starvation was found in *Medicago truncatula* (*Mt4*), *Arabidopsis thaliana* (*IPS1*, *INDUCED BY PHOSPHATE STARVATION1* and *At4*), tomato (*Lycopersicon esculentum* L.; *TPSI1*, *TOMATO PHOSPHATE STARVATION-INDUCED GENE 1*), and rice (*Oryza sativa*; *OsPI1*, *ORYZA SATIVA PHOSPHATE-LIMITATION INDUCIBLE GENE 1*) [21–24]. Further analysis indicated that *IPS1* acts as a decoy of miRNA-399 and allows the accumulation of its target gene transcripts [25]. A third intergenic lncRNA called *LDMAR* (*LONG-DAY-SPECIFIC MALE-FERTILITY-ASSOCIATED RNA*) is required for normal pollen development in rice under long day conditions [26]. A number of lncRNAs are differentially expressed under stress stimuli in *Arabidopsis* [9]. Despite limited reports on the mechanisms of plant lncRNA function, it is evident that plant lncRNAs play vital roles in developmental and stress responses. This realization combined with the advent of next-generation sequencing has prompted tremendous efforts and investments in identifying lncRNAs in a wide range of organisms [5, 27–35].

*F. vesca*, the woodland strawberry, is becoming a new model organism for both octoploid cultivated strawberry (*Fragaria x ananassa*) and other members of the *Rosaceae,* a family that includes many fruit trees. *F. vesca* has a short life cycle, small stature, facile transformation, and small and sequenced genome (2*n* = 14, 240Mb) [36–38]. Further, transcriptomic data of various tissues in the cultivated strawberry were available [39]. Of significant interest is the strawberry fruit, which is developed from the receptacle (the stem tip that supports the flower) [40]. Toward the identification of molecular mechanisms of strawberry floral and fruit development, we first generated comprehensive RNA-seq datasets from 35 distinct *F. vesca* floral and fruit tissues at different developmental stages to profile genome-wide expression of PC genes [41, 42]. In current work, we seek to identify lncRNAs from strawberry floral and fruit transcriptomes with the goal of uncovering lncRNAs that function in flower and fruit development. In total, 5,884 lncRNAs derived from 3,862 loci were identified from the *F. vesca* flower and fruit trancriptome dataset. Further analysis indicated that these lncRNAs are similar to PC genes in terms of gene structure and transcriptional regulation. However, lncRNAs also show a number of distinctions from PC genes. For example, a large number of lncRNAs are precursors to small RNAs, and their sequences are much less conserved than PC genes. In an effort to identify potential regulatory target genes of lncRNAs, we used expression correlation between lncRNAs and the PC genes in the *F. vesca* genome, taking advantage of the RNA-seq data from a large number of different floral and fruit tissues. Both positive and negatively correlated lncRNA-PC gene pairs are identified. Our analysis provided the first look at the lncRNA landscape in a fruit crop and laid the foundation for future studies of lncRNA function in strawberry. To facilitate the study of lncRNAs and data sharing, our *F. vesca* lncRNAs can be accessed from the Strawberry Genome Resources (SGR) website [43] as a new track in gBrowse (http://bioinformatics.towson.edu/strawberry/) as well as from Genome Database for Rosaceae (GDR; https://www.rosaceae.org) [44].

## Results

### Identification of *F. vesca* lncRNAs from flower and fruit RNA-seq datasets

**Fig. 1:**
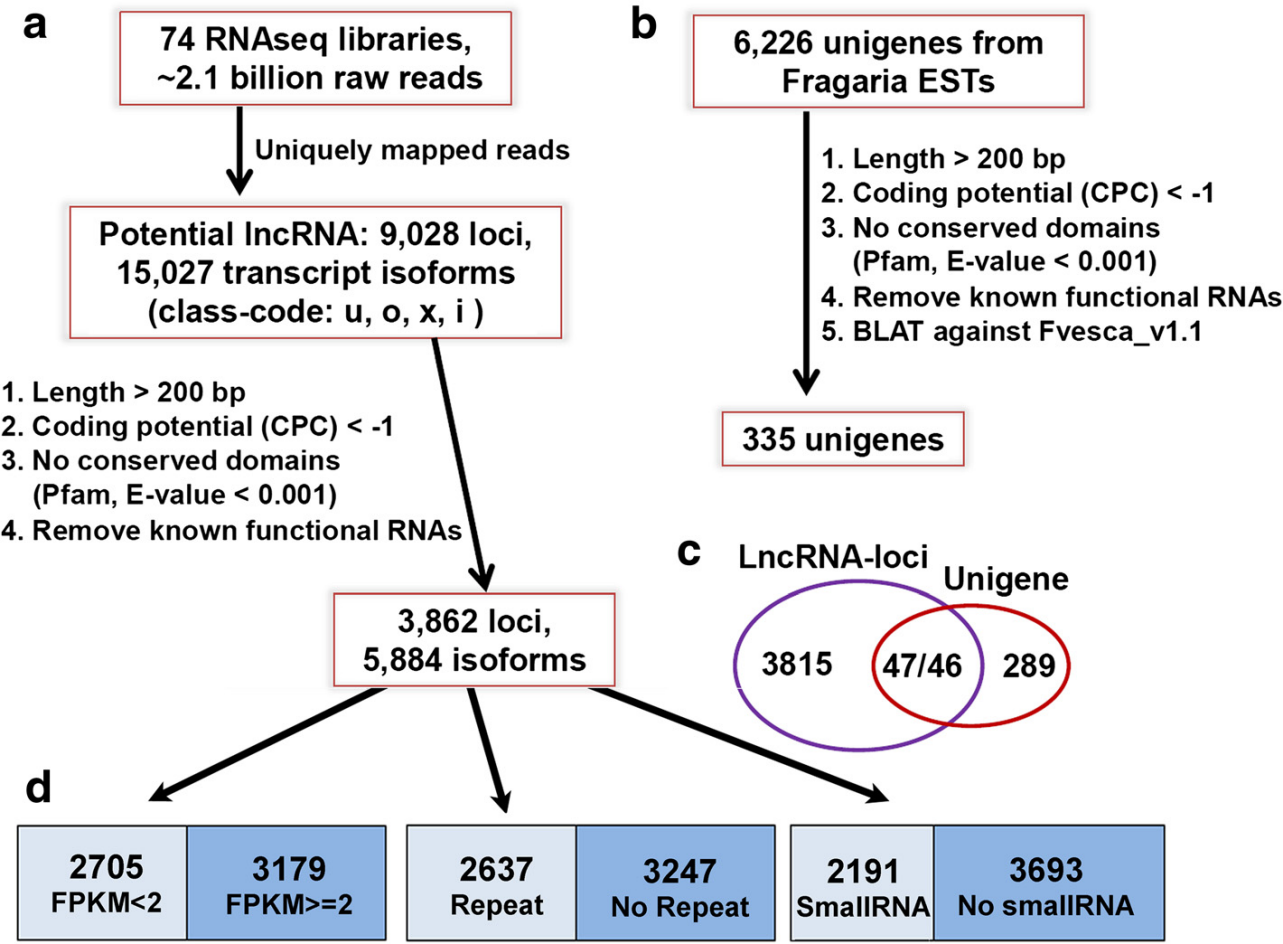
Summary of the workflows used for identifying strawberry lncRNAs. **a** lncRNA identification from *F. vesca* RNA-seq datasets. **b** lncRNA identification from unigenes. **c** Venn diagram comparing lncRNAs identified in (**a**) and (**b**). **d** Different classifications of *fve*-lncRNAs based on expression levels, repeats, and precursors of small RNAs. hc-lncRNAs: high confidence lncRNAs

To globally identify lncRNAs in the *F.vesca* genome, we utilized RNA-seq datasets generated from 35 distinct floral and fruit tissues plus two vegetative tissues (seedlings and young leaves), which were isolated from a 7^th^ generation inbred line of *F. vesca* (Yellow Wonder 5AF7 or YW5AF7) (Additional file 2) [41, 42]. There were two biological replicates for each tissue type, and hence 74 RNA-seq libraries in total, amounting to about 2.1 billion total single-end reads of 51bp. The analysis pipeline is shown in Fig. 1. In brief, each library was aligned individually by *Tophat2* in order to preserve junction reads. Known and novel transcripts were assembled by *cufflinks* based on uniquely mapped reads and, finally, all assemblies were combined by *cuffmerge* and then compared to the annotations by *cuffcompare* to characterize each transcript.

In total, 15,027 candidate noncoding transcripts (8,709 loci) in class “u” (unknown, intergenic), “o” (generic exonic overlap with a reference transcript), “x” (overlap with a reference transcript in the opposite orientation), and “i” (intronic) were subjected to further filtering (Fig. 1a, Table 1). The transcripts are defined as lncRNAs if they are longer than 200bp, are non-coding, and are not a member of known functional RNA classes (tRNA, rRNA, snRNA, miRNA) (Fig. 1a). A relatively stringent non-coding standard was used. First, the *Coding Potential Calculator* was used to discriminate non-coding from coding transcripts as it takes into account several biologically meaningful sequence features with high accuracy [45]; a non-coding transcript has a coding potential score (CPC) lower than “-1”. Second, to reduce false positive, any short peptide coded by “non-coding transcript” should contain no conserved protein domains (Pfam database). As a result, 5,884 transcript isoforms in 3,862 loci were identified as lncRNAs (Additional files 3, 4 and 5).

**Table 1:** The number of lncRNAs in different classes before and after filtering

	Isoform		Locus	
Class-code	Before^a^	After^b^	Before^a^	After^b^
u (unknown or intergenic region)	7,203	3,763	4,726	2,575
o (overlapped with existed gene with a dramatic difference in gene structures)	5,125	625	2,824	464
x (overlapped with existed gene in an opposite direction)	2,658	1,471	1,438	902
i (located in introns)	41	25	40	24
Total	15,027	5,884	8,709^c^	3,862^c^

Notes:

^a^The number of original isoforms and loci predicted by Cufflinks

^b^The number of lncRNAs after filtering

^c^“Total” number is smaller than the sum of the column, because certain loci have multiple isoforms with different class codes

In addition to the RNA-seq data, we mined existing ESTs from *Fragaria*. A large number of raw EST datasets derived from both diploid *Fragaria vesca* and octoploid *Fragaria × ananassa* were found in the NCBI dbEST database, but unigenes from GDR (Genome Database for *Rosaceae*) were chosen for lncRNA discovery as unigenes do not contain low quality ESTs or redundant ESTs [46]. The filtering criteria were similar to the lncRNA discovery from RNA-seq data (Fig. 1b) and led to the identification of 335 lncRNAs out of 6,226 unigenes (Additional files 6 and 7). Interestingly, only 46 of the 334 lncRNAs are in common with the RNA-seq-derived lncRNAs (Fig. 1c). We then compared the remaining 289 unigene-derived lncRNAs with the *F. vesca* genome annotation and found that a majority of them are from annotated PC loci on either the same or opposite strand sometimes covering the introns. A lack of over-lap between RNA-seq- and unigene-derived lncRNAs suggests that the identification of lncRNAs is far from saturation, partly due to a lack of strand orientation information from our RNA-seq reads.

### Identification of high confidence (hc)-lncRNAs

The 5,884 lncRNAs identified from the *F. vesca* RNA-seq data were further characterized based on expression levels and whether or not they contain repeats or generate small RNAs (Fig. 1d; Additional file 8). First, 3,179 lncRNAs are expressed at higher than 2 FPKM (Fragments Per Kilobase of exon per Million fragments mapped) in at least one of the tissue types in both replicates; the remaining 2,705 lncRNAs are expressed at lower than 2 FPKM. Second, lncRNAs that overlapped with transposable elements and/or repeats were identified by RepeatMasker and RepeatScout, respectively (see Methods). In total, 3,247 transcripts with a percentage of repetitive sequences lower than 10 % were classified as “no repeat” (Fig. 1d; Additional file 8).

The filtering pipeline (Fig. 1a) has removed known pre-miRNA transcripts, but a small quantity of the remaining lncRNAs may still encode previously unknown miRNAs. Moreover, some lncRNAs would generate short hairpin RNAs and siRNAs involved in epigenetic regulation [30, 47]. To distinguish those small RNA-generating lncRNAs, we used the small RNA-seq dataset from nine strawberry tissue types [48]. In total, 224 million small RNA-seq reads between 18bp and 30bp were mapped against the 5,884 lncRNAs by Bowtie 1. 7.7 % of the small RNA reads mapped perfectly to the lncRNAs; the majority (58 %) of these small RNAs were 21bp in length. This is in sharp contrast with the total small RNA reads with 24 bp small RNA as the most abundant species (Additional file 1: Figure S1). We thus identified 2,191 potentially small RNA-generating lncRNAs, defined as having higher than 10 small RNA reads mapped to the lncRNA locus (Fig. 1d; Additional file 8).

When all three filtering criteria were applied, only 1,556 high confidence lncRNAs (hc-lncRNAs) were obtained (Additional file 8). They are expressed at a relatively high level (>2 FPKM), contain no repeats, and do not produce small RNAs.

### Characterization of strawberry lncRNAs

The 32,831 predicted PC genes from the *F. vesca* genome are evenly distributed across chromosomes (Additional file 1: Figure S2A). This is in contrast to some other genomes, such as maize and soybean, which have lower gene densities in the pericentromeric regions. Like the PC genes of *F. vesca*, 5,884 *Fve*-lncRNAs are also distributed evenly across the seven chromosomes (Additional file 1: Figure S2A). However, *Fve*-lncRNAs also exhibit marked differences from *Fve*-PC genes. First, lncRNAs have fewer exons (Additional file 1: Figure S2B). The majority of *Fve*-lncRNAs (65 % of lncRNAs and 75 % of hc-lncRNAs) possess only one or two exons, while only 38 % of the PC genes have < = 2 exons. Second, lncRNAs are generally shorter than PC transcripts (Additional file 1: Figure S2C). Third, a larger number of PC genes are expressed at a higher level than lncRNA-coding loci, according to the FPKM extracted from the output of a single *cuffdiff* run (Additional file 1: Figure S2D).

### LncRNAs are expressed in specific tissues and stages

**Fig. 2:**
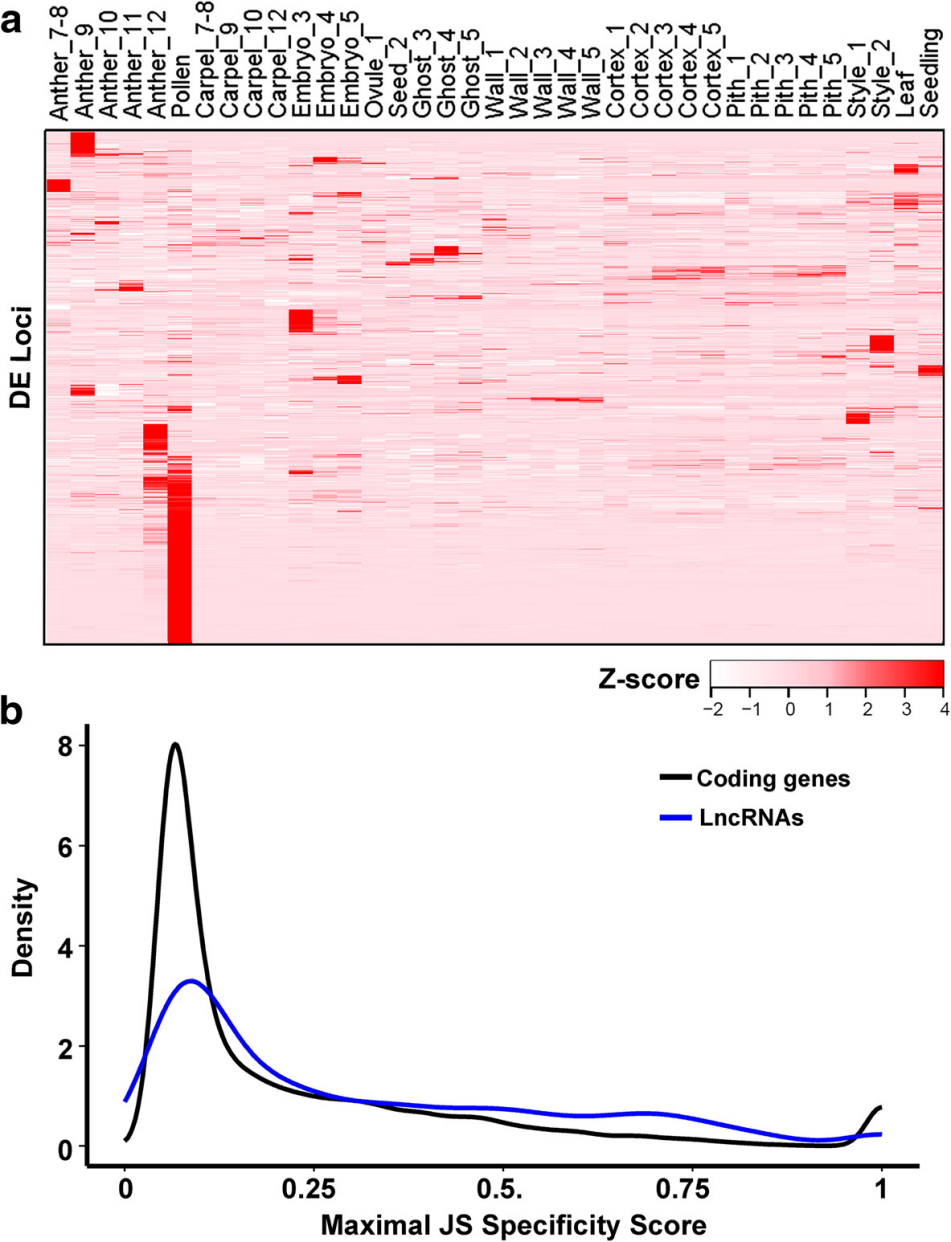
Heatmaps showing tissue-specific expression of differentially expressed lncRNAs. **a** Heatmap showing expression patterns of 1,619 differentially expressed lncRNAs. They are defined as differentially expressed when q-value < 0.01, fold change > 2 in one of the pairwise comparisons. Z-score obtained from averaged FPKM of two replicates was used. **b** The distributions of the maximal JS (Jensen-Shannon) specificity score of coding genes and lncRNA-loci, respectively

During the *cuffdiff* run, both differentially expressed (DE) loci and their isoforms (alternatively spliced transcripts at each locus) were examined by pairwise comparisons between successive developmental stages of the same tissue types (q-value < 0.01, fold change >2, Additional files 9 and 10). In these comparisons, anthers and embryos have the most DE lncRNA isoforms and loci; very few DE lncRNAs were found in receptacle cortex and pith (see details in the Additional files 9 and 10). In total, 1617 isoforms from 1619 loci showed differential expression between at least two different tissue types. The Z-score was obtained for each of these DE loci based on averaged FPKM of two biological replicates. A Z-score-based heatmap was made by hierarchical clustering across all tissues (Fig. 2a). Overall, a large number of lncRNAs were specific to one tissue at one specific stage. The biggest cluster of loci was exclusively expressed in the mature pollen. Relatively more lncRNAs were uniquely expressed in Anther_9, Anther_12, and Embryo_3. It may be that Anther_12 and mature pollen share some specifically expressed lncRNAs since pollen was collected from stage 12 anthers. A similar gene expression trend was also observed when looking at the expression of all isoforms (Additional file 1: Figure S3). Hence, lncRNA expression is spatially and temporally regulated. The JS (Jensen-Shannon) specificity score was used to estimate the degree of tissue specificity. When a gene is expressed exclusively in a particular tissue, its JS score equals to 1. The distribution of the JS score shows that more lncRNAs have a higher score than PC genes (*P* < 2.2 × 10^−16^, Kolmogorov-Smirnov test), suggesting that a higher percentage of lncRNAs were more exclusively expressed (Fig. 2b).

**Fig. 3:**
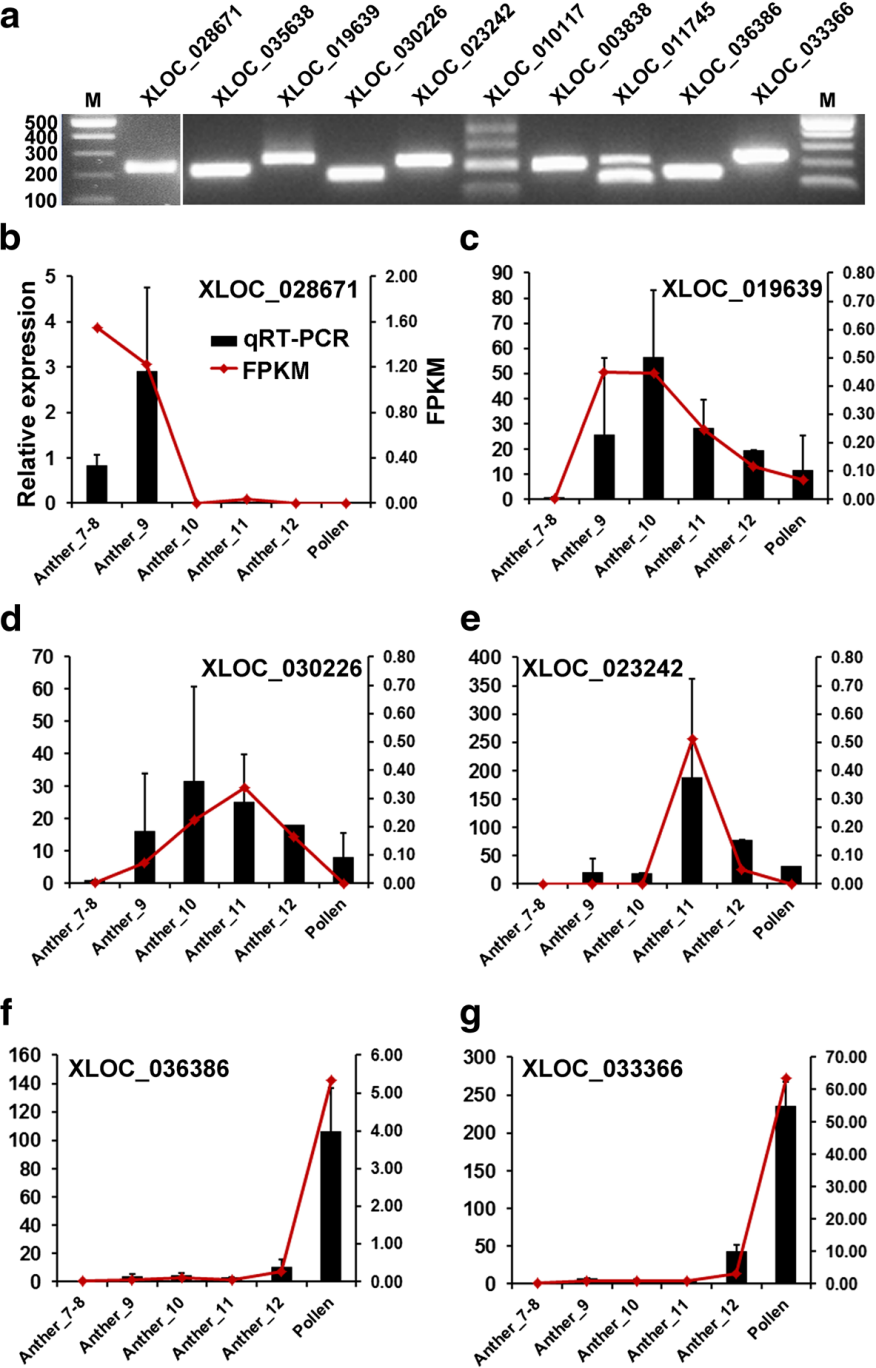
Validation of anther/pollen specific expression of lncRNAs by qRT-PCR. **a** Gel image of RT-PCR products of ten randomly selected anther/pollen specific lncRNAs. **b** to **g** The expression of six lncRNAs quantified by qRT-PCR (black bar and Y-axis on the left). Error bar indicates standard deviation (SD) of two biological replicates with three technical replicates each. The relative FPKM of the same six lncRNAs based on RNA-seq data was also shown (red line and Y-axis on the right). Gene11892 was used as the internal control for both RNA-seq (red line) and qRT-PCR (black bar). RNAs were from anthers at stage7/8, stage9, stage10, stage11, and stage12 as well as mature pollen

To validate the RNA-seq data, ten lncRNAs with anther- or mature pollen-specific expression were selected for verification by RT-PCR (Additional file 11). These lncRNAs have a class_code “u” or “x” with FPKM starting from 4. The expected amplicons were observed for all ten lncRNAs (Fig. 3a), but some primers also amplified non-specific bands in the same tissue (XLOC_010117 and XLOC_011745) or different bands between tissues (XLOC_035638 and XLOC_003838). We further tested the expression levels of six lncRNAs by quantitative RT-PCR. XLOC_028671 is highly expressed in stage 7–9 anthers, just before or at the stage of microspore tetrad formation (Fig. 3b). XLOC_019639 and XLOC_030226 are more abundant in stage 10 anthers, the stage at which the tapetum cells start to degenerate (Fig. 3c, d). XLOC_023242 is highly expressed in stage 11 anthers (Fig. 3e). Both XLOC_036386 and XLOC_033366 are predominantly expressed in mature pollen and slightly expressed in stage 12 anthers (Fig. 3f, g). In general, the qRT-PCR results (black bars) are consistent with the RNA-seq results estimated by Cufflinks (red lines) (Fig. 3).

### Expression correlation of lncRNAs with neighboring PC genes

**Fig. 4:**
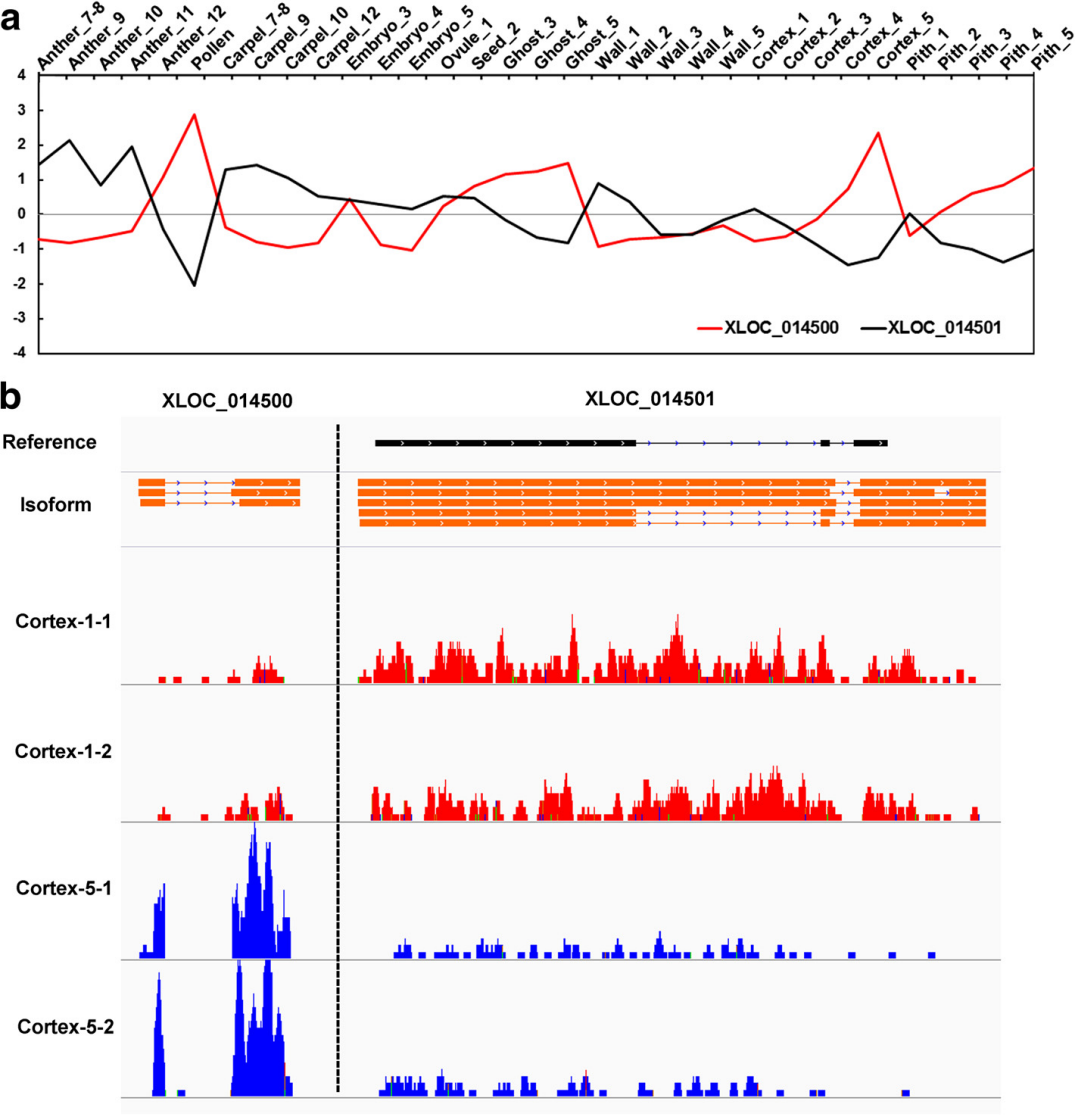
An example of negative correlation in expression between an lncRNA and its neighboring PC gene. **a** The expression of lncRNA XLOC_014500 (red line) and neighboring coding gene XLOC_014501 (black line, gene22438) is negatively correlated. Y-axis shows the expression level by Z-score obtained from averaged FPKM of two replicates. **b** IGV view of aligned RNA-seq read counts for XLOC_014500 and XLOC_014501 based on two fruit tissue stages: Cortex-1 (pre-fertilization) and Cortex-5 (post-fertilization). The panel of “Reference” shows the gene structure based on genome annotation version1.1. Thin line indicates intron and thick line denotes exon. The panel of “Isoform” shows transcript variants predicted by Cufflinks. The bottom four panels illustrate the RNA-seq read counts in respective tissues. The two replicates are shown with identical color

LncRNAs regulate gene expression via a number of mechanisms including the regulation of neighboring loci in *cis* [8, 49]. We thus examined the correlation in expression between lncRNAs and their respective neighboring PC genes. Since the location of lncRNAs in the unanchored pseudo-molecule of the *F. vesca* genome is ambiguous, those lncRNAs were not included in the analysis. 2,870 out of 3,099 lncRNA loci have PC neighbors either upstream or downstream within a 10 kb distance. 1,461 gene pairs showed absolute value of correlation coefficient > 0.5 (Additional file 12) and are thus promising candidates for *cis*-regulation. Although lncRNAs were slightly more positively correlated with neighboring coding genes than PC genes with their neighbors in statistics (Kolomogorv-Smirnov (KS) test, *P* < 0.05; Additional file 1: Figure S4), the distributions of correlation coefficients for lncRNA-to-neighbor and PC-to-neighbor are quite similar, in agreement with findings in human [28]. Further, many more lncRNA-to-neighbor gene pairs showed a positive correlation than those showing a negative correlation (1,422 pairs with r > 0.5 versus 39 pairs with r < −0.5). However, an in depth examination of negatively correlated lncRNA-neighbor pairs revealed some intriguing findings. As shown in Fig. 4, XLOC_014500, an intergenic lncRNA locus with three isoforms and two exons, has a correlation coefficient of −0.645 with the neighboring gene XLOC_014501 (gene22438), which codes for a pentatricopeptide repeat-containing protein. The opposite expression pattern is evident in all 37 tissues (Fig. 4a) and is illustrated in detail for the fruit cortex tissues (Fig. 4b). The lncRNA gene XLOC_014500 is highly expressed in the stage 5 cortex, while XLOC_014501 is lowly expressed in the stage 5 cortex. In contrast, XLOC_014500 is lowly expressed in the stage 1 cortex, while XLOC_014501 is highly expressed in the stage 1 cortex. While the observed expression correlations between *fve*-lncRNAs and their co-expressed neighbors are highly intriguing, whether they reflect true regulatory relationships or not require further testing.

### Expression correlation of lncRNAs with PC genes *in trans*

**Fig. 5:**
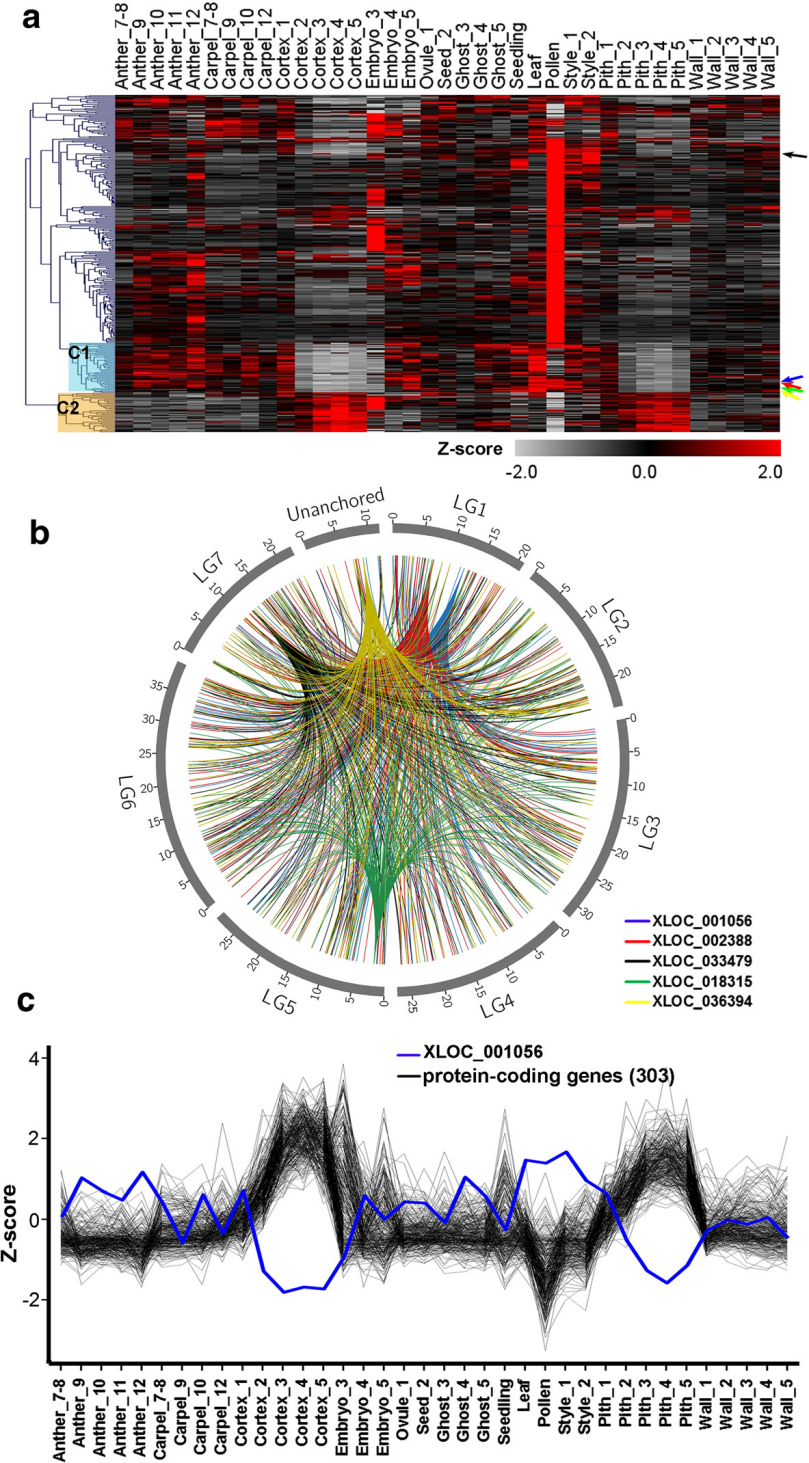
Negative correlation in expression between lncRNAs and PC genes across the genome. **a** Heatmap showing the expression of 313 lncRNAs, which were identified as having 10 or more negatively correlated PC genes across the genome at a cut-off of r < −0.7. Clusters C1 and C2 were highlighted and are specifically repressed or activated in the receptacle. **b** Top five lncRNAs with the highest number of negatively correlated protein coding genes are shown. Each of the five lncRNAs is connected to its negatively correlated protein–coding genes with lines. The expression pattern of each of the five lncRNAs is marked in (**a**) by arrows with the same colors. **c** The opposite expression trend between lncRNA XLOC_001056 (blue line) and its 303 negatively correlated PC genes (black lines). Z-score used in (**a**) and (**c**) was obtained from averaged FPKM from two replicates

Prior reports in animal systems also suggest *trans*-acting modes of some lncRNAs [12, 50]. To identify lncRNAs that may act *in trans*, the expression correlations between lncRNAs and all PC genes in the *F. vesca* genome were calculated. 1,330 out of 3,099 lncRNAs were negatively correlated with PC genes (r < −0.7, Additional file 13). 313 lncRNAs each showed negative coefficients with more than 10 genes; the expression patterns of these 313 lncRNAs are shown in the heatmap (Fig. 5a). While a majority of these lncRNAs showed high levels of expression in the pollen, two clusters of lncRNAs (C1 and C2) showed complementary expression patterns. One cluster (C2 in Fig. 5a) was specifically and more abundantly expressed in the receptacle fruit (cortex and pith) at post fertilization stages (stages 2–5). The complementary cluster (C1 in Fig. 5a) was expressed in other tissues excluding the receptacle (cortex and pith) at stages 2–5. Successful fertilization of ovules has been shown to induce receptacle fruit initiation [40, 51], therefore the C2 cluster of lncRNA genes may be induced by fertilization with potential roles in promoting fleshy fruit initiation. In contrast, the C1 cluster of lncRNAs may possess opposite roles and may be involved in repressing receptacle fruit development.

The five lncRNAs with the highest number of negatively co-regulated genes are shown in a graph (Fig. 5b). Each of the five lncRNAs is connected to the co-regulated genes in the genome with colored lines. These 5 lncRNAs are all expressed at a low level in the receptacle fruit, with four belonging to the C1 cluster (Fig. 5a, Additional file 1: Figure S5A). In contrast, their negatively correlated 553 PC genes are more highly expressed in the receptacle (Additional file 13). Fig. 5c illustrates the expression of lncRNA XLOC_001056 and its corresponding 303 negatively co-regulated PC genes. Given that these 5 lncRNAs could potentially contribute to the regulation of such a large number of negatively correlated PC genes, their impact on the development of fruit could be significant. Enriched GO terms for these 553 PC genes include metabolic processes and intracellular transport processes, consistent with functions in fruit tissue development (Additional file 1: Figure S5B, Additional file 13). These analyses not only identified *fve*-lncRNAs with potential regulatory roles *in trans*, but also highlighted specific lncRNAs that could potentially serve as hubs in a coordinated gene expression networks underlying receptacle fruit development.

### Examination of evolutionary conservation of lncRNAs

If lncRNAs perform evolutionarily conserved functions, they could be conserved across species even though a lack of coding constraints may enable rapid changes in DNA sequences. Thus, we investigated if any of the *fve*-lncRNAs identified in this study are conserved across different plant species. First, we used the *fve*-lncRNAs to blast against the genomes of *Arabidopsis thaliana*, maize (*Zea mays*), rice (*Oryza sativa*), apple (*Malus domestica*)*,* and peach (*Prunus persica*); 36, 50, 52, 450 and 511 *Fve*-lncRNAs were found to share similarities with certain sequences in the respective plant genomes (E-value < 0.001). Since apple, peach, and strawberry belong to the *Rosaceae* family, they are more closely related to one another. The numbers above suggest that about 1 % of the *fve*-lncRNAs have potential conserved counterparts in the non-*Roseceae* species and about 10 % *fve*-lncRNAs have potential homologs in the *Roseceae* species. Hence, the evolutionary conservation of lncRNA is rather limited.

The above analysis could not determine if the homologous sequence in the other species encode lncRNAs. Therefore, we investigated if the *fve*-lncRNAs are homologous to lncRNAs already identified in these species. Currently, 6,480 lncRNAs were identified in *Arabidopsis* [31], 2,224 lncNRAs were identified in rice [35], and several thousand lncRNAs were reported from two studies in *Zea mays.* Boerner and McGinnis first identified 2,492 maize lncRNAs [30]; Li reported 1704 high confidence lncRNAs and 18,459 pre-lncRNAs in maize [47]. 5,884 fve-lncRNAs were blasted against the lncRNAs from *Arabidopsis*, rice and maize, only one fve-lncRNA (TCONS_00042468, class_code “u”) was found to share similarity to the pre-lncRNA transcript TCONS_00012579 in maize (Table 2).

**Table 2:** Identification of conserved lncRNAs in related species

Number of *Fve-*lncRNAs with homologs in other species	Number of *Arabidopsis* lncRNAs with homologs in other species	Total lncRNAs in other species	Other Species	Reference for lncRNA-identification in other species
0/0	-/-	6,480	*Arabidopsis*	Liu et al. [31]
0/0	0/0	2,492	Maize	Boerner and McGinnis [30]
0/0	0/0	1,704	Maize (hc-lncRNAs)	Li et al. [32]
1/1	5/27	18,459	Maize (pre-lncRNAs)	
0/0	5/9	2,224	Rice	Zhang et al. [35]
-/-	0/0	5,884	*Fragaria vesca*	This wrok
49/37	2/2	4,301	*Malus*-unigene	This work
19/7	0/0	1,315	*Prunus*-unigene	This work

Notes:

Column 1 shows the number of *Fve*-lncRNAs with homologs in other (non-*Fragaria*) species listed in column 4. The number before ‘/’ refers to lncRNA number in *Fragaria* and the number after ‘/’ refers to the number of lncRNA in the target (non-*Fragaria)* species. The numbers in column 1 were derived from blast searches using strawberry lncRNAs as queries against the target species. The blast E-value cutoff is < 0.001. The second column shows results using *Arabidopsis* lncRNAs as queries against corresponding target species. No blast was performed against the query itself and hence was marked as ‘-/-’

To test conservation of *fve*-LncRNAs in the *Rosaceae* species, *Malus* and *Prunus*, we took advantage of available ESTs for these two species. We first identified lncRNAs from each species applying the same filtering criteria as for *F. vesca*. Specifically, we analyzed 25,525 assembled unigenes in the *Malus* unigenes v.5 downloaded from GDR; these unigenes were built previously based on ESTs from mainly *Malus domestica*. Among them, 4,301 unigenes were found to possess lncRNAs (Additional file 14). 37 of these *M. domestica* unigenes exhibited sequence similarities to 49 *F. vesca* lncRNAs (E-value < 0.001) (Table 2). Similarly, analysis of 10,934 assembled unigenes in the *Prunus* unigenes v.5 (based on ESTs from apricot, peach, Chinese plum, and cherry) yielded 1,315 unigenes that likely produce lncRNAs (Additional file 15). 7 of these *Prunus* unigenes showed sequence similarity to 19 *F. vesca* lncRNAs (E-value < 0.001) (Table 2). The conservation of these lncRNAs within the same plant family suggests that these lncRNAs may arise in the common ancestor of these species and may confer biological functions unique to this family of plants.

To investigate possible conservation of lncRNAs across any plant species described above, the 6,480 lncRNAs from *Arabidopsis* [31] were blasted against the lncRNAs from maize, rice, *Malus* and *Prunus*, respectively. Five of these *Arabidopsis* lncRNAs showed similarity to 27 of 18,459 pre-lncRNAs from maize (E-value < 0.001) [47]. Five *Arabidopsis* lncRNAs are similar to 9 of the 2,224 lncRNAs from rice (E-value < 0.001) [35]. Two *Arabidopsis* lncRNAs are similar to 2 of the 4,301 lncRNAs from *Malus* (E-value < 0.001). No homolog was identified in other blasts. Therefore, lncRNAs were not well conserved in higher plants, perhaps reflecting the fast evolving nature of lncRNAs due to the lack of constraint normally imposed upon PC genes [5, 52, 53].

## Discussion

LncRNAs are being increasingly recognized as an important class of regulatory molecules in both animals and plants. While lncRNAs have been widely studied in human and animals, they are still poorly studied in plants with the exception of a limited number of model plant species. To date, no lncRNA has been described in strawberry nor in any other *Rosaceae* species. In this study, we performed genome-wide identification of lncRNAs from diploid strawberry *Fragaria vesca* as well as several other *Rosaceae* species including *Malus* and *Prunus*, thereby providing a first look at the landscape of noncoding genes in the *Rosaceae* genomes. Since *F. vesca* possesses a full complement of molecular genetic tools, the discovery of fve-lncRNAs laid the groundwork for future functional studies of lncRNAs.

Since *Rosaceae* is an important family for fruit crops, the regulation of their flower and fruit development is of considerable interests. Previously we developed extensive floral and fruit tissue transcriptomes for *F. vesca* via RNA-seq and small RNA-seq [41, 42, 48]. In this study, we mined these transcriptome datasets for lncRNAs using a set of stringent filtering criteria. We identified 5,884 *F. vesca* lncRNAs coded by 3,862 loci. We showed that these *F. vesca* noncoding loci possess features similar to PC loci; they contain promoters, exons, and introns, and are alternatively spliced. On the other hand, we showed that the lncRNAs are not well conserved when compared with PC genes, have fewer exons, are expressed at lower levels, and are shorter in transcript length (Additional file 1: Figure S2). These findings are consistent with other studies on plant lncRNAs [29–35]. One most striking feature of these lncRNAs is their tissue-specific expression indicating possible function in specific flower or fruit tissues or at specific stages of development.

The lncRNAs identified from our study may represent only a fraction of lncRNAs coded by the *F. vesca* genome. This is because these lncRNAs were only identified from libraries derived from flower and fruit tissues grown under normal conditions. Further, since the RNA-seq libraries were made from polyA-selected mRNAs, nonpolyadenylated lncRNAs were missed from these libraries. Finally, natural antisense transcripts (NATs) transcribed from the presumptive non-coding DNA strand could be missed due to a lack of strand-specific information in our libraries. Therefore, our work only represents the initial genome scale identification of lncRNAs. Nevertheless, the large number of lncRNAs identified in this study suggests that lncRNAs may contribute significantly to strawberry flower and fruit development.

### Plant lncRNAs are not well conserved during evolution

Blast searches of f*ve*-lncRNAs against the lncRNAs identified in several non *Rosaceae* plant species yielded none or very few homologs (Table 2), indicating limited evolutionary conservation. Though non-coding RNAs evolve faster than PC genes [52], thousands of conserved lncRNAs have been found among primates and several tetrapod species [5, 53], possibly owing to more ancient origin of these lncRNAs found in animals and more time to be stabilized in function. In contrast, flowering plants only arose 130 million years ago and their lncRNAs may be of recent origin and maybe relatively young and transient.

It is not surprising why lncRNAs are not well conserved. First, lncRNAs are not constrained by codon usage. Second, lncRNAs may possess short conserved motifs, but these short motifs are not easily identifiable by BLAST. For example, an intergenic lncRNA family *Mt4*/*IPS1*/*At4*/*TPSI1*/*OsPI1* found in multiple plant species [21–24] contains a ~23 bp short and conserved motif; this motif binds miR-399 via sequence complementarity, the basis of “target mimicry” [25]. Target mimicry is emerging as a prevalent mode of action by lncRNAs since more lncRNAs are found to act through target mimicry [35, 54]. Third, 36 % lncRNAs are probably associated with small RNAs in this study, most of which should be siRNAs (Fig. 1, Additional file 8). The siRNAs could be generated from a short pairing fragment of two RNAs, such as lnc-NATs, or the stem-loop structure of a single RNA, which are less constrained in other parts of the transcripts [55, 56]. Fourth, some lncRNAs may directly interact with RNA-binding proteins through conserved secondary structures [9, 57].

### The expression of lncRNAs is highly tissue-specific

A major challenge in deducing lncRNA function resides in that lncRNAs do not encode proteins. Gene expression, specifically tissue-specific expression, may help shed light on the potential function of these lncRNAs. Pairwise comparisons between tissues or stages revealed that 27.5 % of lncRNA isoforms and 52.3 % of lncRNA loci are differentially expressed and show high tissue specificity (Fig. 2), suggesting that lncRNAs are subject to active transcriptional regulation. Most notably, a large number of lncRNAs are highly and specifically expressed in the mature pollen (Fig. 2). However, PC genes are also highly and specifically expressed in the mature pollen [42]. This similarity may simply reflect that mature pollen is a very unique tissue. Nevertheless, the action of pollen-specific lncRNAs and their interaction with pollen-specific PC genes may underlie the unique characteristics of mature pollen. The precise regulation of lncRNAs in specific tissues support that lncRNAs may play important functions during reproductive development.

### Expression correlations of lncRNAs with PC genes *in cis* and *in trans*

In animals and plants, lncRNAs have been shown to act either *in cis* or *in trans* to regulate PC gene expression [7, 18, 20, 49, 50]. Based on the idea that lncRNAs and their regulatory targets may exhibit highly correlative expression either positively or negatively, we sought to identify potential regulatory targets of the fve-lncRNAs by taking advantages of the available RNA-seq data for a large number (thirty-seven) of *F. vesca* tissues. A similar strategy was successfully employed in finding candidate regulatory targets of lncRNAs in mammals and model plants [5, 8]. Through this strategy, we identified 1,423 positively correlated and 39 negatively correlated pairs of lncRNAs and neighboring genes (Additional file 12, Fig. 5). The positive expression correlations may simply result from their subjecting to common regulations of local chromatin. However, some correlations could reflect authentic regulatory relationships between an lncRNA and its neighboring gene. An encouraging example is the positive expression correlation between lncRNA *APOLO* and its regulatory target *PID*, which codes for a key regulator of polar auxin transport [8]. In our study, we showed an example of an lncRNA XLOC_014500 and its negatively correlated PC gene XLOC_014501 (Fig. 4). XLOC_014501 encodes a protein belonging to the PPR protein family that has RNA binding capacity [58]. The opposite expression pattern and the immediate upstream location of the lncRNA XLOC_014500 with respect to the PPR gene warrants further investigation into a possible direct regulatory relationship between the lncRNA and its neighboring PPR gene.

To identify lncRNAs acting *in trans*, we performed a correlation analysis between 3,112 lncRNAs and all PC genes in the *F. vesca* genome (Additional file 13). One third of the lncRNAs have negatively correlated PC genes at a cut-off of −0.7; this is not surprising given the pervasive *in trans* regulations reported in animals [12, 50]. In examining the expression profiles of these lncRNAs, we identified clusters of lncRNAs exhibiting receptacle fruit-specific repression or activation (Fig. 5a, C1 and C2). It will be interesting to determine if C1 and C2 clusters play opposite roles in the development of this unique strawberry fruit type. Among the lncRNAs that show receptacle-specific repression, we selected 5 that have expression patterns correlated with the highest number of PC genes (Fig. 5b; Additional file 1: Figure S5). The 553 coding genes targeted by these five lncRNAs are enriched in various metabolic processes associated with active cell proliferation and growth (Additional file 1: Figure S5). These results suggest that the receptacle-repressive lncRNAs could serve as the hubs of a gene regulatory network, the repression of which may lead to positive receptacle fruit growth.

## Conclusions

5,884 *Fve*-lncRNAs derived from 3,862 loci were identified from diploid strawberry *Fragaria vesca* using the flower and fruit RNA-seq datasets, thereby providing a first look at the landscape of noncoding genes in one fruit crop of the *Rosaceae* family. The tissue-specific lncRNA expression patterns and the gene expression correlation analysis between lncRNAs and PC genes identified a set of lncRNAs with potential roles in flower and fruit development. The discovery of fve-lncRNAs laid the groundwork for future functional studies of lncRNAs in strawberry.

## Methods

### Description of RNA-seq datasets

Two transcriptome datasets generated from diploid strawberry YW5AF7 [59] during flower and fruit development were used for lncRNA identification here. One dataset includes two biological replicates of 25 samples representing 5 different fruit tissues at 5 developmental stages (Additional file 2) [41]. The second dataset includes two biological replicates each of 12 samples representing developing anthers and carpels of flowers (Additional file 2) [42]. RNA-seq libraries were made from polyA-selected RNA and sequenced using Illumina HiSeq2000. About 20–40 million single-end, 51 bp reads were obtained per sample (Additional file 2). Both datasets were deposited at Sequence Read Archive (SRA) at NCBI (http://www.ncbi.nlm.nih.gov/sra). The accession numbers are SRA065786 and SRP035308, respectively.

### Identification of novel transcripts

Sequence reads of each library were aligned individually to the version 1.1 *F. vesca* genome which has 32,831 annotated PC genes (Fvesca_226.fa and Fvesca_226_gene.gff3 downloaded from http://phytozome.jgi.doe.gov/pz/portal.html) using the TopHat 2.0 program [60]. During the alignment, the minimal anchor length was set as 5 (−a), the maximal intron length was set as 5000 (−I), and other settings were at default. Only uniquely aligned reads were used for further analysis (“NH:i:1”). To derive all novel transcripts, the unique reads were assembled individually using the Cufflinks 2.0 program. Next, transcripts with coverage higher than 2 in each library were combined using Cuffmerge and then compared to Fvesca_226_gene.gff3 to assign the class_code to each transcript using Cuffcompare [61]. Finally, Cuffdiff was used to call all the differentially expressed genes in pairwise comparisons (*q*-value < 0.01, fold change >2). The gene expression level at FPKM (Fragments Per Kilobase of exon per Million fragments mapped) was obtained by the CummeRbund R package, and tissue specificity score (JS score) was calculated for each transcript using the csSpecificity() function in this package.

### Filtering strategy used to identify lncRNAs

Among the assembled transcripts, the majority are partially (72,727, class_code “j”) or completely (26,093, class_code “=”) matched with the existing annotation. As the version 1.1 annotation includes only PC genes, these two categories (j and =) should represent PC genes and were thus excluded from further analysis. The transcripts with class_code “u” (unknown intergenic transcript), “o” (generic exonic overlap with a reference transcript), “x” (natural antisense transcript, NAT), and “i” (intronic transcript) were subjected to PC potential calculation [45]. Non-coding transcripts (coding potential score (CPC) < −1) larger than 200 bp were extracted for further analysis. Transcripts with unknown direction were kept only if both orientations possess no coding potential. Further, transcripts that encode any conserved protein domains were removed in the sense strand for multi-exonic transcripts or in either strand for single-exon transcripts. These transcripts were identified by searching against the Pfam database (E-value < 0.001) [62]. The remaining transcripts were blasted against the Rfam database (http://rfam.xfam.org/), tRNA database (http://gtrnadb.ucsc.edu/), and rRNA database (http://ssu-rrna.org/) to remove any known transcripts (E-value < 0.001). To eliminate all possible pre-miRNAs, transcripts that perfectly match the 362 miRNAs found in the octoploid and diploid strawberries were filtered out [63–65]. To discover lncRNAs from ESTs (*Expressed Sequence Tag*), the fifth version of *Fragaria* unigene downloaded from GDR (www.rosaceae.org) was used for filtering following the same pipeline described above. In addition, they were mapped to the *F. vesca* genome by BLAT with at least 95 % sequence identity in the matched region (−minIdentity) and 50 % matched in length.

### Conservation of lncRNAs

To determine conservation of lncRNAs, fve-lncRNAs were blasted against a few plant genomes and lncRNAs from other species using standalone blastn program (blast-2.2.28+, E-value < 0.001). The genomes of *Arabidopsis* (Arabidopsis_thaliana.TAIR10), maize (Zea_mays.AGPv3), and rice (Oryza_sativa.IRGSP-1.0) were downloaded from the release 28 of the ensemble website (http://plants.ensembl.org/index.html). The genomes of apple (Malus_x_domestica.v3.0.a1) and peach (Prunus_persica_v2.0.a1) were downloaded from GDR. The data resources of lncRNAs used in this study are shown in Table 2. The fifth version of unigenes from the genera of *Malus* and *Prunus* were downloaded from GDR. To discover lncRNAs from *Malus* and *Prunus*, their respective unigenes were similarly filtered (length > 200 bp; CPC < −1). House-keeping RNAs and conserved miRNAs were removed as well.

### Removal of transcripts that can yield small RNAs or contain repetitive sequence

Raw small RNA-seq reads generated from nine tissue types in woodland strawberry YW5AF7 [48, 59] were previously deposited at the Gene Expression Omnibus (GEO) at NCBI under accession numbers GSE44930 and GSE61798. We re-analyzed the raw reads by quality-filtration (quality score –q = 28, percent of bases –p = 80 %), then combined all reads and clipped off the adaptors. The processed reads were collapsed into a single FASTA file by FASTX-Toolkit (http://hannonlab.cshl.edu/fastx_toolkit/). The number of total collapsed reads was 24,405,396. Only reads of 18bp to 30bp in length were used in further analysis. Those reads were aligned to the 5,884 lncRNAs with perfect match by Bowtie1 [66]. The aligned small RNA reads composed of simple repeats or mapped to more than 20 loci were removed. Finally, the lncRNAs were separated into two groups: lncRNAs generating small RNAs (>10 reads per alignment) and lncRNAs not generating small RNAs (<= 10 reads per alignment).

*De novo* prediction of repetitive sequences in putative fve-lncRNAs was achieved by RepeatScout with default parameters [67]. Subsequent similarity searches and repeat masking were performed by RepeatMasker (http://www.repeatmasker.org) against both the repeat library obtained by RepeatScout and the *Rosaceae* repeat library (version 20140131) deposited at the RepBase (http://www.girinst.org/). The lncRNAs were separated into two groups: lncRNAs associated with repetitive sequences (> = 10%) and lncRNAs not associated with repetitive sequences (<10 %).

### Quantitative RT-PCR of lncRNAs

RNAs were isolated from stage7/8 to stage 12 anthers and mature pollen using the RNeasy Plant Mini Kit (Qiagen, cat. No.74904) and the RNase-Free DNase Set (Qiagen, cat. No.79254). Two biological replicates were included. cDNA was synthesized from ~1 μg total RNA in 20μl solution using iScript™ cDNA Synthesis Kit (Bio-Rad Laboratories, Cat. #170-8891). 5× diluted cDNA was used as the template in real-time PCR analysis. SsoFast™ EvaGreen® Supermix (Bio-Rad Laboratories, Cat. #172-5203) was used to set up real-time PCR reactions, which were run and analyzed on the CFX96 Real-Time System (Bio-Rad Laboratories). Conditions for real time PCR were: 98 °C for 3 min, followed by 55 cycles of 95 °C for 15 s, and 60 °C for 10 s. Melting curve analysis was performed from 65 °C to 95 °C with increments of 0.5 °C. Gene-specific primers are listed in Additional file 13. The Pfaffl formula 2^-ΔΔCt^ method was used to calculate relative gene expression differences. Stably expressed gene11892 was used as the internal control [41].

### Correlation analysis between lncRNAs and PC genes

To test the correlation of expression between lncRNAs and their neighboring genes, both upstream and downstream PC genes within 10 kb distance of the 3,099 lncRNAs were identified by Bedtools (makewindows and intersect) [68]. PC genes that overlap with lncRNAs were not included in this analysis. The Pearson correlation coefficient was calculated by cor() using average FPKM of two replicates in R. To obtain the expression correlation of lncRNAs and distant PC genes, correlation coefficients were similarly calculated between lncRNAs and all the other PC genes excluding known neighbor genes. As too many positive correlations were found, only negative correlations with r < −0.7 were preserved. The *p*-value was calculated by the function corPvalueStudent() in the R package WGCNA [69].

## Availability of supporting data

The data sets supporting the results of this article are included within the article and its additional files. Readers can also visualize *F. vesca* lncRNAs as a track in gBrowse hosted at SGR (http://bioinformatics.towson.edu/strawberry/) as well as at GDR (https://www.rosaceae.org).

## Additional files

Additional file 1: Figure S1. Length distribution of small RNAs derived from lncRNAs. **Figure S2.** Characterization of strawberry lncRNAs. **Figure S3.** Heatmaps showing tissue-specific expression patterns of lncRNAs. **Figure S4.** Expression correlations between lncRNAs and the adjacent PC genes. **Figure S5.** Negative correlations of lncRNA expression with PC genes across the genome. (PDF 12315 kb) Additional file 2:
**The descriptions and mapping statistics of the 74 samples.** (XLSX 16 kb) Additional file 3:
**The sequences of 5,884 lncRNAs in the .fasta format.** (FASTA 5602 kb) Additional file 4:
**The annotation file of 5,884 lncRNAs in the .gtf format (cufflink output).** (GTF 3031 kb) Additional file 5:
**Annotations of the 5,884 lncRNAs.** (XLSX 336 kb) Additional file 6:
**The sequences of 335 unigenes in the .fasta format.** (FASTA 223 kb) Additional file 7:
**The annotation file of 335 contigs in the .gff3 format (BLAT output).** (GFF3 19 kb) Additional file 8:
**Categories of the 5,884 lncRNAs classified by expression level, repetitive sequences, and small RNAs.** (XLSX 1624 kb) Additional file 9:
**Differentially expressed lncRNA isoforms in pairwise comparisons.** (XLSX 3558 kb) Additional file 10:
**Differentially expressed lncRNA loci in pairwise comparisons.** (XLSX 2178 kb) Additional file 11:
**Primers used in qRT-PCR for validation of ten random lncRNAs.** (XLSX 8 kb) Additional file 12:
**Expression correlations between lncRNAs and adjacent PC genes.** (XLSX 894 kb) Additional file 13:
**Negative correlation between lncRNA expression and all PC genes.** (XLSX 2974 kb) Additional file 14:
**LncRNAs in** ***Malus.*** (FASTA 2792 kb) Additional file 15:
**LncRNAs in** ***Prunus.*** (FASTA 1011 kb)

## Abbreviations

LncRNA: Long non-coding RNA
Hc-lncRNA: High-confidence lncRNA
PC: Protein coding
siRNA: Small interfering RNA
Lnc-NAT: Long non-coding natural antisense transcript
GDR: Genome Database for Rosaceae
CPC: Coding potential score
FPKM: Fragments Per Kilobase of exon per Million fragments mapped
DE: Differentially expressed
JS score: Jensen-Shannon specificity score
KS test: Kolomogorv-Smirnov test
EST: Expressed Sequence Tag
