# Supplementary material for: Global identification and analysis of long non-coding RNAs in diploid strawberry Fragaria vesca during flower and fruit development
Source: BMC Genomics. 2015 Oct 19;16:815. doi: 10.1186/s12864-015-2014-2 (PMC4617481; doi:10.1186/s12864-015-2014-2)
Supplement: Additional file 1: Figure S1. — Length distribution of small RNAs derived from lncRNAs. Figure S2. Characterization of strawberry lncRNAs. Figure S3. Heatmaps showing tissue-specific expression patterns of lncRNAs. Figure S4. Expression correlations between lncRNAs and the adjacent PC genes. Figure S5. Negative correlations of lncRNA expression with PC genes across the genome. (PDF 12315 kb) [file 12864_2015_2014_MOESM1_ESM.pdf]

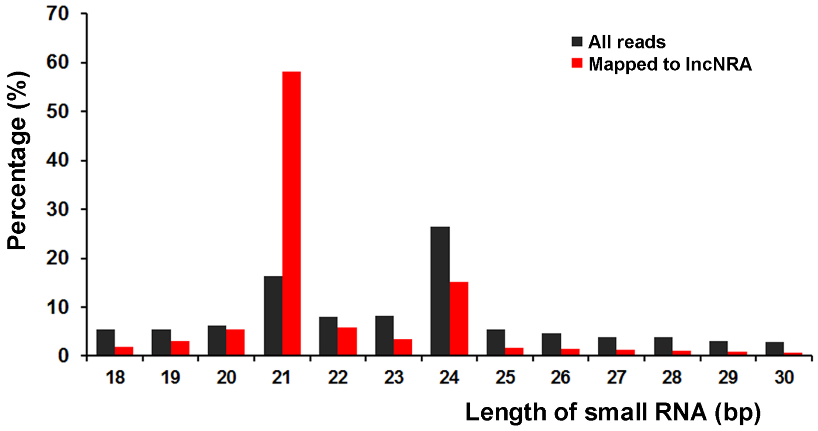


**Figure S1. Length distribution of small RNAs derived from lncRNAs.**

Small RNA reads with a length between 18bp and 30bp were mapped against the 5,884 lncRNAs. "All reads (black bars)" stands for all small RNA reads combined from the nine small RNA-seq libraries. "Mapped to lncRNA (red bars)" stands for small RNA reads that mapped to lncRNAs. LncRNAs derived small RNAs are predominantly 21bp in length, which is in sharp contrast to all small RNAs predominantly 24bp in length.

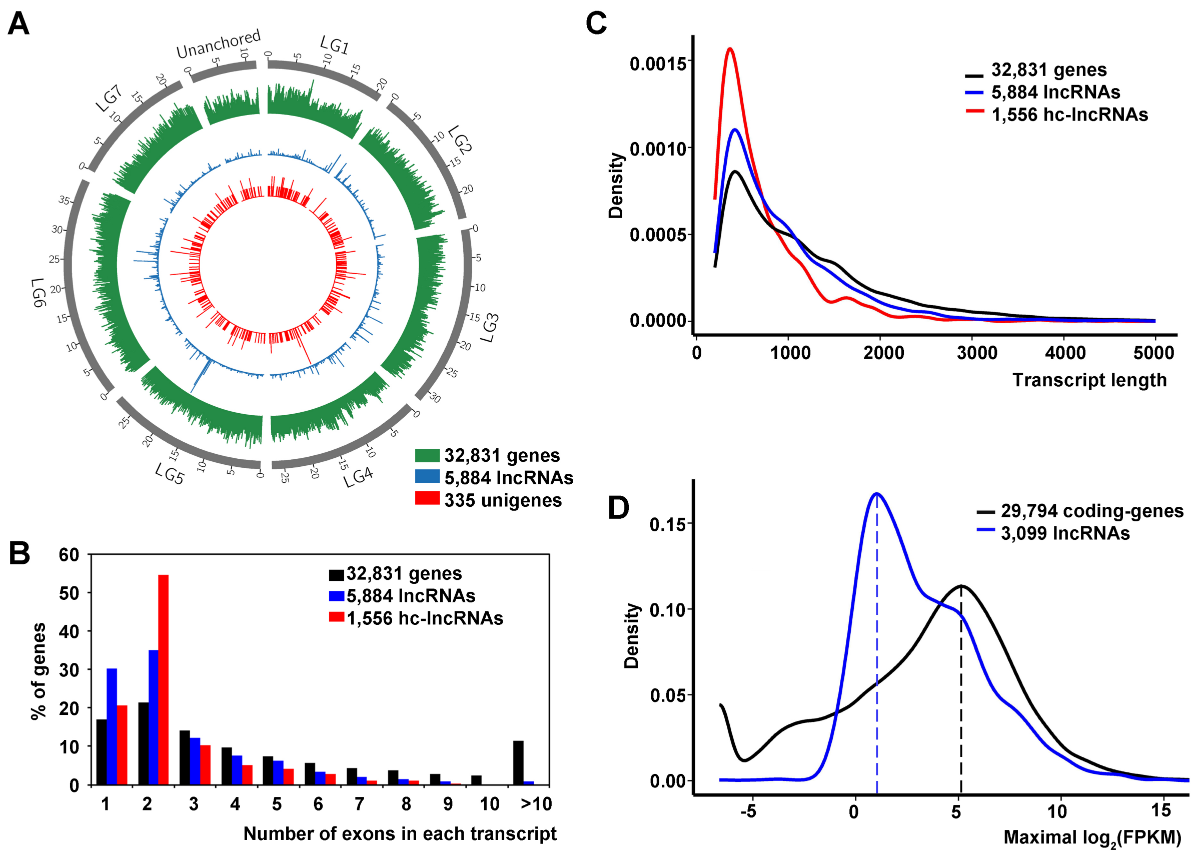


**Figure S2. Characterization of strawberry lncRNAs.**

(A) Distribution of lncRNAs across chromosomes. Histograms of PC genes, lncRNAs from RNAseq and lncRNAs from unigenes were generated by Circos (http://circos.ca/) in a sliding window of 50 kb. (B) Number of exons harbored by PC genes, lncRNAs, and hc-lncRNAs, respectively. (C) Density plot showing the range of transcript length of PC genes, lncRNAs, and hc-lncRNAs, respectively. (D) Density plot showing the range of transcript expression levels of the PC genes and the lncRNA-producing loci. The expression data was obtained from the same run of cuffdiff. Only the highest FPKM of each locus across all tissues was used in (D).

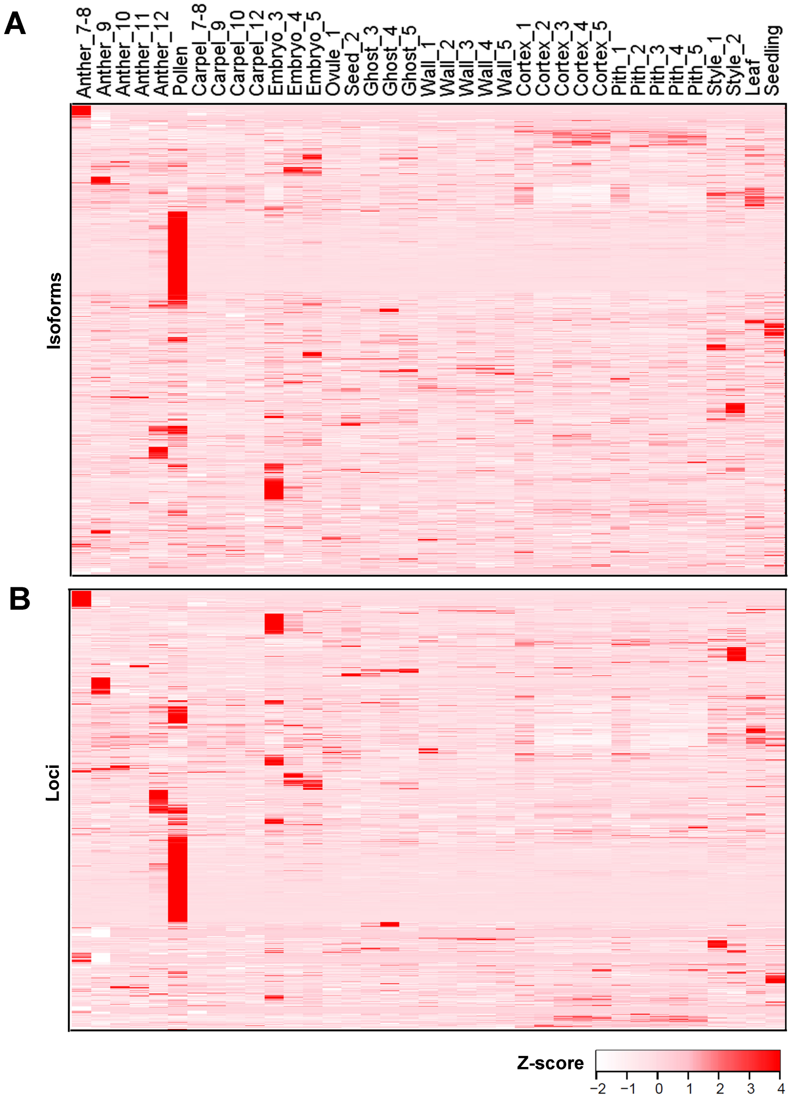


**Figure S3. Heatmaps showing tissue-specific expression patterns of lncRNAs.**

(A) Expression levels of the 5,884 lncRNA isoforms across tissues. (B) Expression levels of the 3,099 lncRNA loci across tissues. Each row is one lncRNA. Mature pollen has the most specifically expressed lncRNAs. Scale bar: Z-score obtained from average normalized FPKM of two biological replicates.

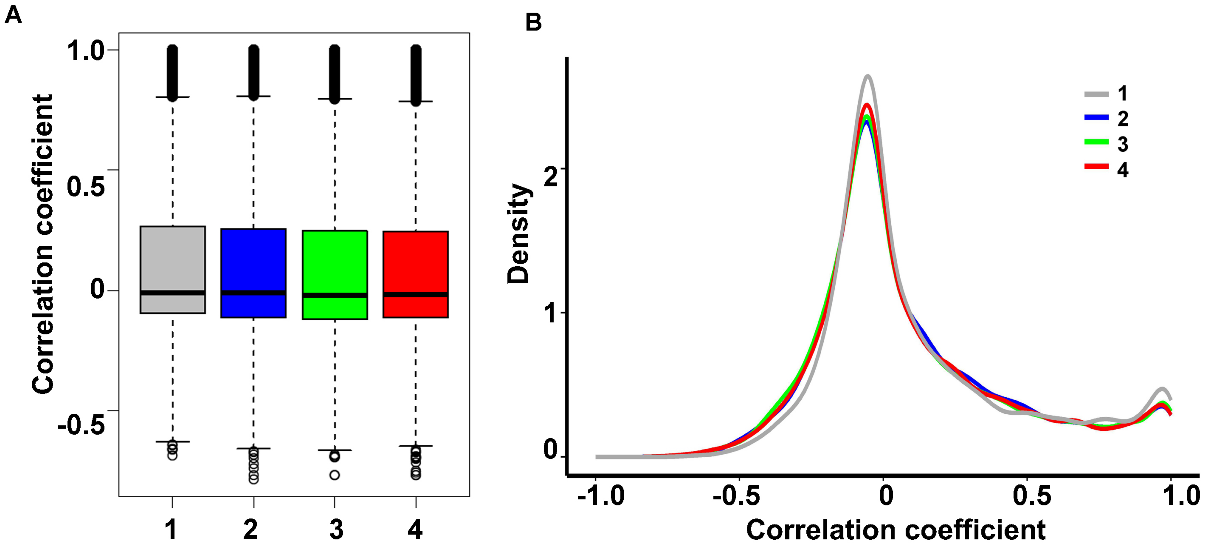


**Figure S4.** **Expression correlations between lncRNAs and adjacent protein-coding genes.** The boxplots (A) and distributions (B) of Pearson correlation coefficients (r) between 3,099 fve-lncRNA loci and their adjacent PC genes (10 kb upstream or downstream, grey line, sample 1) and between 3,099 randomly selected PC genes and their adjacent PC genes with 3 permutations (sample 2-4) were shown. Although lncRNAs are slightly more positively correlated with their PC neighbors than that of coding genes by statistics (Kolomogorv-Smirnov (KS) test, $P < 0.05$), the distributions of correlation coefficients are quite similar to each other.

A

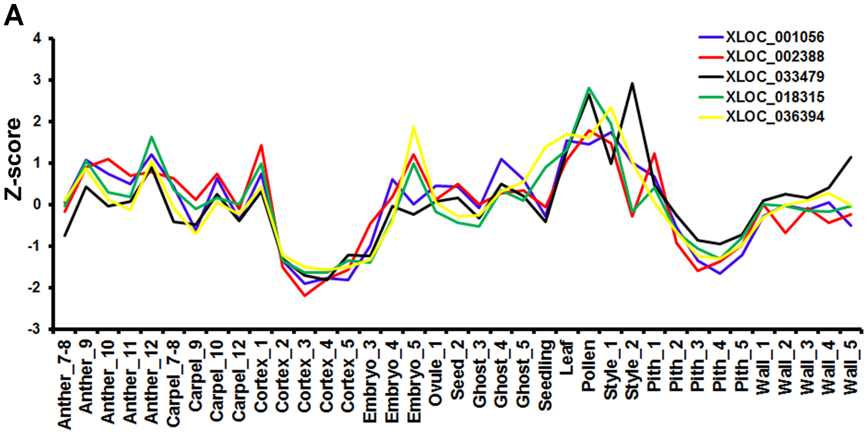


B

| GO-ID | Term | FDR |
|---|---|---|
| GO:0044281 | small molecule metabolic process | 8.67E-08 |
| GO:1901564 | organonitrogen compound metabolic process | 9.18E-07 |
| GO:0055086 | nucleobase-containing small molecule metabolic process | 8.89E-06 |
| GO:0044710 | single-organism metabolic process | 5.54E-05 |
| GO:0009117 | nucleotide metabolic process | 1.07E-04 |
| GO:0006753 | nucleoside phosphate metabolic process | 1.38E-04 |
| GO:1901566 | organonitrogen compound biosynthetic process | 3.39E-04 |
| GO:0006520 | cellular amino acid metabolic process | 8.35E-04 |
| GO:1901605 | alpha-amino acid metabolic process | 1.65E-03 |
| GO:0009116 | nucleoside metabolic process | 2.76E-03 |

**Figure S5. Negative correlations of lncRNA expression with PC genes across the genome.** (A) The expression pattern of the top 5 lncRNAs shown in Fig. 5B. (B) Top 10 enriched GO terms for the 553 PC genes, whose expression is negatively correlated with these top 5 lncRNAs shown in (A) (FDR < 0.01).
